# Rasch Analysis of the Korean Version of the Tinnitus Handicap Inventory

**DOI:** 10.3390/jcm12185785

**Published:** 2023-09-05

**Authors:** Ga-Young Kim, Young Sang Cho, Ji Hyun An, Jung-Wan Kim, Il Joon Moon

**Affiliations:** 1 Hearing Research Laboratory, Samsung Medical Center, Seoul 06351, Republic of Korea; kgyslp@gmail.com (G.-Y.K.); divetosun@gmail.com (Y.S.C.); 2 Medical Research Institute, Sungkyunkwan University School of Medicine, Suwon 16419, Republic of Korea; 3 Department of Digital Health, Samsung Advanced Institute for Health Sciences & Technology, Sungkyunkwan University, Seoul 06355, Republic of Korea; 4 Department of Otorhinolaryngology-Head and Neck Surgery, Sungkyunkwan University School of Medicine, Samsung Medical Center, Seoul 06351, Republic of Korea; 5 Department of Psychiatry, Sungkyunkwan University School of Medicine, Samsung Medical Center, Seoul 06351, Republic of Korea; sereinjh@gmail.com; 6 Department of Speech and Language Pathology, College of Rehabilitation Sciences, Daegu University, Gyeongsan 38453, Republic of Korea; thfrj@daum.net; 7 Samsung Advanced Institute for Health Sciences & Technology, Sungkyunkwan University, Seoul 06355, Republic of Korea

**Keywords:** tinnitus, Rasch analysis, Tinnitus Handicap Inventory

## Abstract

Tinnitus is the perception of abnormal sounds in the ears or head without external auditory stimulation. While classical test theory is often used in tinnitus questionnaire development, it has limitations in assessing item characteristics. Item response theory (IRT) offers more precise individual ability estimations and identifies key and less important items, making it superior for reliable measurement tools. This study investigated the suitability of the Korean version of the Tinnitus Handicap Inventory (K-THI) as a patient-reported outcome measure (PROM) for clinical trials. Using Rasch analysis based on IRT, we evaluated K-THI’s measurement of tinnitus-related disability in 545 patients (40.4% men, 59.6% women). Five items (2, 7, 8, 19, and 24) did not fit the Rasch model, yet a unidimensional scale and good fit for person and item data emerged (person: 0.89; item: 0.98). The three-point rating scale in K-THI proved suitable. IRT allowed precise evaluation of K-THI’s properties, vital for reliable PROMs in patient-centered care. Our findings highlight IRT’s role in questionnaire development, contributing to the advancement of PROMs.

## 1. Introduction

Tinnitus is the perception of abnormal sounds in the ears or head without any external auditory stimulation. The prevalence rate of tinnitus ranges from 8 to 25.3% in the United States [1], and there is a strong correlation between subjective tinnitus and various causes of hearing loss [2]. In Korea, the prevalence of tinnitus among adults aged 20 and above in the past year was as high as 20.7% [3]. Recently, an increasing number of patients with tinnitus have begun seeking medical intervention to identify the underlying cause and receive treatment options to improve their quality of life (QoL).

Self-reported questionnaires are commonly used to assess tinnitus because subjective distress is considered more important to its treatment than psychoacoustic measurements such as pitch matching and loudness matching [4]. Most tinnitus is subjective symptom, generally perceived only by the individual experiencing it, without being noticeable to others. Therefore, self-reported questionnaires offer an effective means of evaluating the effects of tinnitus on an individual’s QoL, including the severity of the symptoms, the level of distress, and the impact on daily activities. 

Classical test theory (CTT) assumes that every observed score consists of two components: a true score and an error score [5]. The true score represents an individual’s actual level of ability, and the error score accounts for random factors that could influence the observed score but are not related to the true score. CTT focuses on the overall performance of a test and assumes that a person’s observed score reflects both the true score and error score. However, despite its widespread use, CTT has some limitations. For instance, it assumes that the error score is random and unrelated to the true score, which might not always be accurate. Furthermore, CTT does not consider test-taking strategies, such as strategic omission, which can affect test scores. Those limitations have prompted the development of alternative measurement theories, such as item response theory (IRT).

IRT models the relationship between a person’s ability level and the difficulty level of the test items [6]. A Rasch analysis is a type of IRT model used to estimate the probability that an item will receive a correct response based on the level of the underlying construct being measured and the difficulty level of the item. The main goal of a Rasch analysis is to transform raw scores into a linear scale that reflects the level of the underlying construct being measured. 

The terms “ability” and “difficulty” are used generically in a Rasch analysis to describe what is being measured about persons and items, respectively. When reporting Rasch results, the terminology is tailored to match the specific construct being measured [7]. For instance, person ability refers to the level of the construct being measured by a questionnaire, and item difficulty describes the level of challenge posed by the items on the questionnaire. 

CTT assumes that all the items on a questionnaire are equally difficult, and IRT assumes that the items on a questionnaire have different levels of difficulty. This means that IRT provides more detailed information about an individual’s ability level and the characteristics of the items being measured than CTT. IRT is particularly useful for identifying misfitting items, detecting item bias across different subgroups of a population, and improving the overall quality of a questionnaire. By accounting for differences in item difficulty, IRT can provide a more accurate estimation of a person’s ability than CTT and identify which items are contributing the most or the least to the measurement of the underlying construct. 

Most tinnitus questionnaires have been developed using CTT, which can be problematic because it does not examine the item characteristics in detail. The Tinnitus Handicap Inventory (THI) is the most commonly used self-report questionnaire for evaluating tinnitus [8]. It has been cross-culturally adapted and translated into several languages, including Mandarin [9], Danish [10], and Korean [11]. However, these questionnaires have some limitations due to their development based on CTT. 

Our purpose in this study was to evaluate the measurement properties of the K-THI using a Rasch analysis to determine its suitability as a patient-reported outcome measure (PROM) for high-quality clinical trials. By using a Rasch analysis, we sought to acquire a more detailed understanding of the K-THI’s item characteristics and its ability to distinguish between individuals with different levels of tinnitus severity. 

## 2. Materials and Methods

### 2.1. Participants

We retrospectively analyzed K-THI data collected from patients who visited the outpatient Department of Otolaryngology at Samsung Medical Center between January 2022 and March 2023. The inclusion criteria for this study were (1) age 19 years or older, (2) reporting tinnitus as their primary complaint, and (3) completing the K-THI. A minimum of 200 participants is required for a Rasch analysis [12], and we obtained 545 data points for our study. Among the 545 participants, 220 (40.4%) (54.26 ± 14.03 years) were men, and 325 (59.6%) (54.60 ± 13.55 years) were women. Approval from the institutional review board (IRB) was obtained prior to conducting this study (SMC 2023-03-073-001).

### 2.2. Tinnitus Handicap Index

The THI is a questionnaire used to assess the effects of tinnitus on an individual’s daily life. It consists of 25 items that measure the severity of tinnitus across three domains: functional (11 items), emotional (9 items), and catastrophic (5 items). The rating scale used in the THI consists of three response options: “No” (0 points), “Sometimes” (2 points), and “Yes” (4 points), with a higher score indicating a greater level of patient handicap resulting from tinnitus [8]. The questionnaire is widely used in clinical and research settings to evaluate the effectiveness of tinnitus interventions and measure changes in tinnitus severity over time.

### 2.3. Statistical Analysis

The Rasch analysis is a formal testing method used to evaluate the compatibility of a measure with the Rasch model, as originally proposed by Rasch [13]. The primary objective of a Rasch analysis is to evaluate the degree to which observed responses differ from predicted responses. If observed responses fall within an acceptable range of the predicted responses, then the data are said to fit the model, and they are considered to be unidimensional and invariant and provide interval levels of measurement [14,15,16], indicating that the questionnaire generates values that can be classified as measurements and allowing for great precision and accuracy in assessments.

When questionnaires, such as the THI, contain items that have more than two possible scores, a mathematical version of the Rasch model for polytomous data is used instead of the dichotomous model. In this study, the Rasch analysis was performed using WINSTEPS^®^ software version 5.4.2 (Winsteps, Chicago, IL, USA) and based on the Rasch–Andrich rating scale model [17]. The specific properties investigated as part of a Rasch analysis are detailed below [7].

### 2.4. Dimensionality and Item Fit Statistics

To assess the dimensionality of the K-THI, we used three methods. The first method used Rasch fit values to identify misfit items. Misfitting items were identified as those with outfit or infit mean square (MNSQ) values greater than 1.4 or less than 0.6, which indicate a need to revise or remove those items from the test to improve its validity and reliability. An MNSQ value greater than 1.4 indicates that an item is underfit, i.e., it does not measure the same construct as the other items. An MNSQ value of less than 0.6, on the other hand, suggests that an item is overfit, i.e., the model predicts data better than expected but it might not harm the model.

The second method involved conducting a Rasch factor analysis using the principal component analysis method of residuals (PCAR) to detect secondary dimensions in the data. This analysis used two criteria to verify unidimensionality: (1) more than 40% of the variance must be explained by the Rasch dimension, and (2) an eigenvalue less than 2 for the 1st contrast is considered noise, but an eigenvalue of 3 suggests the presence of systematic variance and a potential second dimension. These criteria were used to ensure that the questionnaire measures a single construct and that the items function appropriately.

Third, we created an item–person map, a visual tool used in a Rasch analysis to illustrate how the items in a questionnaire relate to the abilities of the people being assessed. The items are ranked by their level of difficulty based on the frequency of reports, with the most commonly reported (easiest) item located at the bottom and the least reported (most difficult) items at the top. The map provides a way to assess how well the items in a questionnaire align with the abilities of the people being assessed, which is a crucial factor in determining the quality of PROMs. 

Rasch person and item reliability indices were used to indicate the replicability of the person and item placements along the trait continuum. The person reliability index estimates the consistency of person placements that can be expected if the same individuals were given another set of items that measured the same construct. Meanwhile, the item reliability index estimates the consistency of item placement within a hierarchy of items along the measured variable, assuming that the same items are given to another sample of comparable ability. The indices are similar to Cronbach’s alpha and range from 0 to 1, with higher values indicating greater reliability. In this study, both the person and item reliability indices had a value above 0.81, which indicates good reliability and suggests that the results are likely to be replicable. 

### 2.5. Rating Scale

The rating scale diagnostics for the K-THI were determined based on three criteria. First, average measures were used for the vertical arrangement of the rating scale, with the lowest score at the bottom and the highest score at the top. Second, the outfit MNSQ values were checked, and those less than 2.0 were considered acceptable. Finally, the difference in thresholds between adjacent categories was examined, with values between 1.0 and 5.0 logits considered appropriate. 

## 3. Results

### 3.1. Dimensionality and Item Fit Statistics

**Figure 1:**
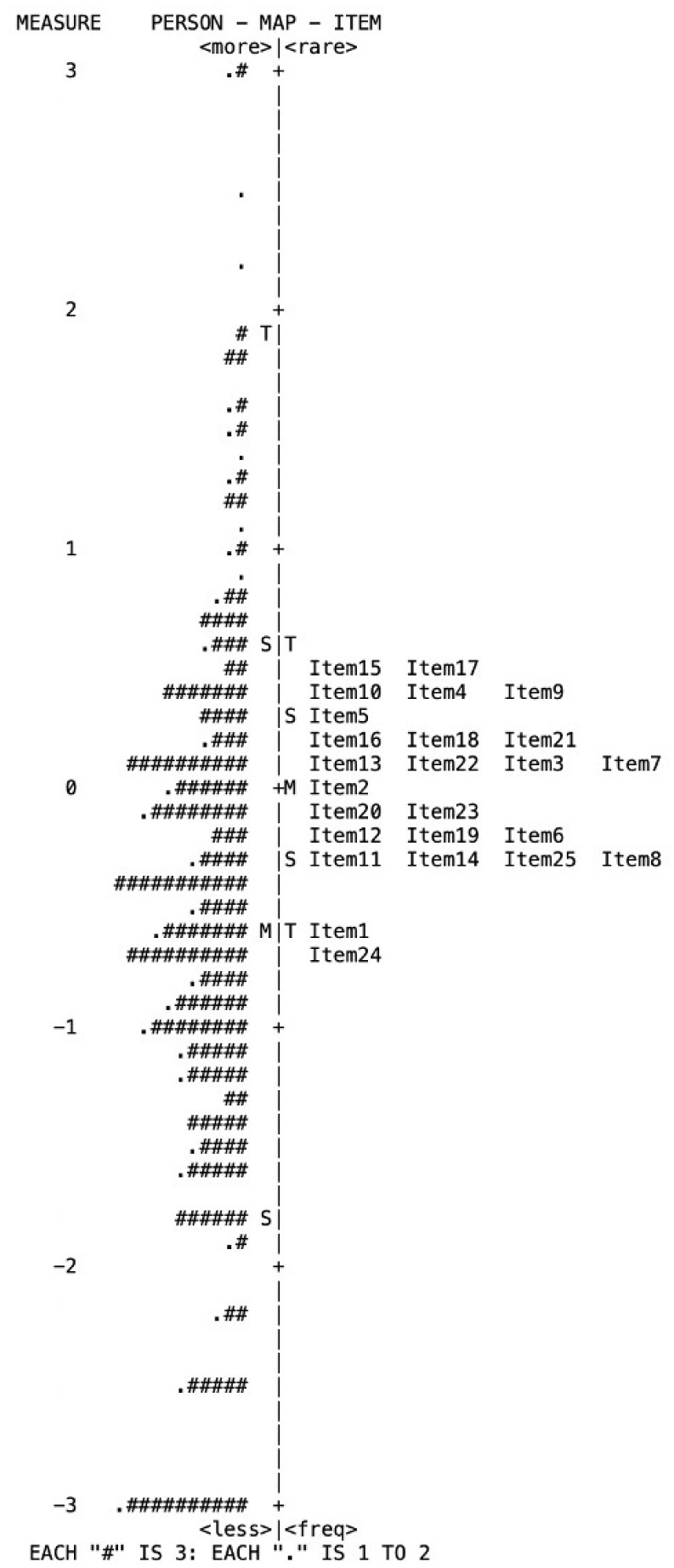
Item–person map for the Korean version of the Tinnitus Handicap Inventory.

Of the 25 items, 5 (no. 2, 7, 8, 19, and 24) were identified as misfits based on item fit statistical analysis because of underfit values (MNSQ values greater than 1.4) (Table 1). In the dimensionality analysis with PCAR, the Rasch dimension explained 50.2% of the variance, and >40% is considered a strong measurement of dimension (Table 2). An item–person map is shown in Figure 1. In this study, item 24 (“Does your tinnitus get worse when you are under stress?”) reflected the symptom most commonly reported by patients. On the other hand, items 15 (“Because of your tinnitus, is it difficult for you to read?”) and 17 (“Do you feel that your tinnitus problem has placed stress on your relationships with members of your family and friends?”) reflected the symptoms least frequently reported by patients. 

### 3.2. Person and Item Reliability

The fit statistics show that both person and item ability had a good fit to the Rasch model, with a reliable estimate of 0.89 for person and 0.98 for item (Table 3).

### 3.3. Rating Scale

The THI uses a three-point rating scale with a category order of zero, two, and four. The average measure scores were found to increase consistently as the category increased. The goodness-of-fit statistics were all within an acceptable range, with the outfit MNSQ value being less than 2.0. The Rasch–Andrich threshold also demonstrated monotonic progression, with the difference in thresholds between adjacent categories ranging from 1.0 to 5.0 logits (Table 4). 

## 4. Discussion

This study used a modern psychometric analysis to evaluate the K-THI as a PROM for high-quality clinical trials in tinnitus management. The THI has previously been criticized for its unclear factor structure and uncertain ability to accurately measure aspects of tinnitus severity [18]. However, our study findings support the use of the K-THI as a reliable unidimensional scale for evaluating tinnitus.

We identified five items with high MNSQ values, indicating that they did not align well with the overall pattern of the K-THI questionnaire [7]. A previous study suggested removing items 2, 8, 13, 19, and 24 due to poor model fit [18], and our study similarly found that items 2, 7, 8, 19, and 24 did not fit the model well. One possible explanation for these results is that patients might have varying attitudes toward tinnitus that could influence their responses to those items. For instance, items 8 and 19 are related to catastrophic feelings and might be perceived differently by individuals based on their cognitive vulnerability.

Although five items did not fit the model well, dimensionality analysis revealed that the Rasch dimension explained 50.2% of the variance in the data, and 40% is considered to indicate a strong measurement of dimension [19]. Our study suggests that the K-THI is a unidimensional scale, meaning that all the items measure the same underlying construct. This finding is consistent with previous research conducted in Poland [18]. The THI is designed to assess an overall sense of discomfort related to tinnitus, rather than measuring the discomfort associated with specific individual areas. Social and emotional discomfort are interconnected and have organic relationships in reality [11].

One significant advantage and practical application of the Rasch analysis is its ability to provide a detailed analysis of individual items through the item–person map [7,18]. In this study, items with low locations, such as “Does your tinnitus get worse when you are under stress?” (Item 24) and “Because of your tinnitus, is it difficult for you to concentrate?” (Item 1), indicate the symptoms most commonly reported by patients. Given that many previous studies have reported that tinnitus can develop or worsen after experiencing high levels of stress or that stress can exacerbate existing tinnitus [20,21], our findings are consistent with existing research.

Conversely, the two least frequently reported items were “Because of your tinnitus, is it difficult for you to read?” (Item 15) and “Do you feel that your tinnitus problem has placed stress on your relationship with members of your family and friends?” (Item 17). The low reporting frequency for item 15 can be attributed to the fact that reading is not a hobby for many people. Additionally, patients with tinnitus in this study reported few difficulties in their social relationships, which could be due to the support and understanding they receive from their family and friends [22].

Rasch analysis can measure a type of reliability that is similar to the reliability measured through CTT using Cronbach’s alpha coefficient, which includes person and item reliability. In our study, we found that both person and item reliability were high, with person reliability at 0.89 and item reliability at 0.98 [7,18]. These findings suggest that the instrument is dependable and produces consistent results.

Another advantage of using a Rasch analysis is that it can evaluate the effectiveness of the rating scales used in questionnaires, which is not possible with traditional CTT. In our study, we used Rasch analysis to assess the three-point rating scale in the K-THI questionnaire. The results show that the rating scale is within an acceptable range, indicating that the use of a three-point rating scale in the K-THI is appropriate.

In conclusion, the growing emphasis on patient-centered care highlights the need for reliable data from PROMs. Our use of IRT allowed us to accurately assess the measurement properties of the K-THI, which can provide clinicians and researchers with increased confidence in their diagnoses and trial results based on the K-THI. It is worth noting that the use of modern psychometric analyses, such as the Rasch model, for questionnaire development is not yet widespread in the medical field. However, the benefit of using IRT models instead of CTT includes the ability to gain more detailed information about the properties of the questionnaire being developed. Therefore, our study could contribute to the advancement of PROMs by highlighting the advantages of using IRT models for questionnaire development.

This study has limitations that should be addressed in future research. Although the THI is a reliable and valid scale, its scoring can be influenced by subjective difficulties. Therefore, it is important to consider the sociodemographic characteristics and emotional state of the participants when interpreting the results. However, due to the retrospective nature of this study, it was challenging to verify those aspects because only the participants’ age and sex were available. Tinnitus is not a simple symptom, but rather a condition accompanied by emotional aspects. In a future study, it will be necessary to consider the sociodemographic characteristics and emotional state associated with this condition.

## Figures and Tables

**Table 1:** Item statistics for the Korean version of the tinnitus handicap index.

No.	Item	Logit	SE	Infit		Outfit		Point Measure
				MNSQ ^a^	ZSTD	MNSQ ^a^	ZSTD	
1	Because of your tinnitus, is it difficult for you to concentrate?	**−0.60**	**0.04**	0.97	−0.41	1.05	0.66	0.70
2	Does the loudness of your tinnitus make it difficult for you to hear people?	−0.01	0.05	**1.46**	6.74	**1.51**	5.72	0.60
3	Does your tinnitus make you angry?	0.10	0.05	0.83	−2.92	0.82	−2.25	0.71
4	Does your tinnitus make you feel confused?	0.42	0.05	1.19	2.84	1.17	1.61	0.62
5	Because of your tinnitus, do you feel desperate?	0.26	0.05	0.96	−0.58	0.91	−0.99	0.70
6	Do you complain a great deal about your tinnitus?	−0.17	0.04	0.90	−1.72	0.87	−1.83	0.72
7	Because of your tinnitus, do you have trouble falling asleep at night?	0.13	0.05	**1.44**	6.47	**1.59**	5.99	0.60
8	Do you feel as though you cannot escape your tinnitus?	−0.31	0.04	**1.46**	6.81	**1.55**	6.51	0.63
9	Does your tinnitus interfere with your ability to enjoy your social activities (such as going out to dinner, to the movies?)	0.37	0.05	1.04	0.60	1.03	0.33	0.68
10	Because of your tinnitus, do you feel frustrated?	0.37	0.05	0.78	−3.81	0.70	−3.39	0.73
11	Because of your tinnitus, do you feel that you have a terrible disease?	−0.30	0.04	1.07	1.11	1.09	1.25	0.70
12	Does your tinnitus make it difficult for you to enjoy life?	−0.21	0.04	0.67	−6.21	0.63	−5.72	0.78
13	Does your tinnitus interfere with your job or household responsibilities?	0.06	0.05	0.98	−0.35	0.97	−0.34	0.70
14	Because of your tinnitus, do you find that you are often irritable?	-0.29	0.04	0.65	−6.77	0.64	−5.65	0.77
15	Because of your tinnitus, is it difficult for you to read?	0.51	0.05	0.95	−0.85	0.88	−1.06	0.69
16	Does your tinnitus make you upset?	0.22	0.05	0.72	−5.09	0.67	−4.18	0.75
17	Do you feel that your tinnitus problem has placed stress on your relationships with members of your family and friends?	0.45	0.05	0.90	−1.62	0.81	−1.92	0.70
18	Do you find it difficult to focus your attention away from your tinnitus and on to other things?	0.22	0.05	0.77	−4.04	0.71	−3.64	0.74
19	Do you feel that you have no control over your tinnitus?	−0.24	0.04	**1.51**	7.43	**1.54**	6.42	0.62
20	Because of your tinnitus, do you often feel tired?	−0.14	0.04	0.80	−3.49	0.76	−3.46	0.74
21	Because of your tinnitus, do you feel depressed?	0.19	0.05	0.76	−4.23	0.72	−3.62	0.74
22	Does your tinnitus make you feel anxious?	0.05	0.05	1.00	0.00	1.01	0.18	0.70
23	Do you feel that you can no longer cope with your tinnitus?	−0.10	0.05	1.29	4.51	1.20	2.52	0.67
24	Does your tinnitus get worse when you are under stress?	−0.73	0.04	1.30	4.63	**1.49**	5.23	0.64
25	Does your tinnitus make you feel insecure?	−0.27	0.04	0.79	−3.78	0.78	−3.29	0.75
	Mean	0.00	0.05	1.01	−0.19	1.00	−0.20	

MNSQ = mean square; SE = standard error; ZSTD = Z-standard. ^a^ MNSQ values outside the range of 0.6 to 1.4 were identified as misfits (boldface).

**Table 2:** Standardized residual variance.

Variance	Eigenvalue	Observed (%)	Expected (%)
Total raw variance in observations	50.22	100	100
Raw variance explained by measures	25.22	50.2 ^a^	50.6
Raw variance explained by persons	21.05	41.9	42.3
Raw variance explained by items	4.16	8.3	8.4
Raw unexplained variance (total)	25	49.8	49.4
Unexplained variance in 1st contrast	2.73 ^b^	5.4	10.9

This analysis used two criteria to verify unidimensionality: ^a^ >40% of the variance is explained by the Rasch dimension, and ^b^ the eigenvalue of the 1st contrast is lower than 2.

**Table 3:** Person and item summary statistics.

	Reliability ^a^	Separation	Mean Measure	Model SE	Infit		Outfit	
					MNSQ	ZSTD	MNSQ	ZSTD
Person	0.89	2.84	37	24.8	1.01	0.0	1.00	0.0
Item	0.98	6.55	806	154.1	1.01	−0.2	1.00	−0.2

MNSQ = mean square; SE = standard error; ZSTD = Z standard. ^a^ A value greater than 0.81 for both indices indicates good reliability.

**Table 4:** Diagnostic for rating scale.

Category Score	Observed Count (%)	Average Measure ^a^	Infit MNSQ	Outfit MNSQ ^b^	Andrich Thresholds ^c^
0	5578 (41)	−1.25	1.00	1.01	None
2	6017 (44)	−0.23	0.97	0.94	−1.70
4	2029 (15)	0.82	1.06	1.09	1.70

MNSQ = mean square. ^a^ Average measure scores increased monotonically as the category increased. ^b^ Outfit MNSQ has to be less than 2.0. ^c^ Threshold calibration should progress monotonically, and the difference in thresholds between adjacent categories was between 1.0 and 5.0 logits.

## Data Availability

The data that support the findings of this study are available upon reasonable request.
